# Correlation Study and Predictive Modelling of Ergonomic Parameters in Robotic-Assisted Laparoscopic Surgery

**DOI:** 10.3390/s24237721

**Published:** 2024-12-03

**Authors:** Manuel J. Pérez-Salazar, Daniel Caballero, Juan A. Sánchez-Margallo, Francisco M. Sánchez-Margallo

**Affiliations:** 1 Bioengineering and Health Technologies Unit, Jesús Usón Minimally Invasive Surgery Centre, ES-10071 Cáceres, Spain; mjperez@ccmijesususon.com (M.J.P.-S.); dcaballero@ccmijesususon.com (D.C.); 2 Scientific Direction, Jesús Usón Minimally Invasive Surgery Centre, ES-10071 Cáceres, Spain; msanchez@ccmijesususon.com

**Keywords:** minimally invasive surgery, robotic surgery, robotic-assisted surgery, ergonomics, motion analysis, predictive model, artificial intelligence

## Abstract

BACKGROUND: This study aims to continue research on the objective analysis of ergonomic conditions in robotic-assisted surgery (RAS), seeking innovative solutions for the analysis and prevention of ergonomic problems in surgical practice. METHODS: Four different robotic-assisted tasks were performed by groups of surgeons with different surgical experiences. Different wearable technologies were used to record surgeons’ posture and muscle activity during surgical practice, for which the correlation between them was analyzed. A predictive model was generated for each task based on the surgeons’ level of experience and type of surgery. Two preprocessing techniques (scaling and normalization) and two artificial intelligence techniques were tested. RESULTS: Overall, a positive correlation between prolonged maintenance of an ergonomically inadequate posture during RAS and increased accumulated muscle activation was found. Novice surgeons showed improved posture when performing RAS compared to expert surgeons. The predictive model obtained high accuracy for cutting, peg transfer, and labyrinth tasks. CONCLUSIONS: This study expands on the existing ergonomic analysis of the lead surgeon during RAS and develops predictive models for future prevention of ergonomic risk situations. Both posture and muscle loading are highly related to the surgeon’s previous experience.

## 1. Introduction

Laparoscopic robotic-assisted surgery (RAS) has grown rapidly over the past decades and has become a standard in several surgical procedures [1]. Although RAS has well-known advantages, such as precision in the performance of surgical procedures, three-dimensional visualization of the surgical field, or reduced hospital stays [2], it remains a physically and mentally demanding technique for surgeons. These limitations can be detrimental to surgeons’ health and have an impact on the quality of surgical procedures and patient care [3,4]. Studies reflect that 56.1% of regularly practicing robotic surgeons continue to experience related physical symptoms or discomfort, including neck stiffness, finger fatigue, and eye fatigue, among the most common [5]. Although ergonomic conditions are considered to be improved in RAS compared to conventional laparoscopic surgery, mainly for the lead surgeon operating from the console, scientific evidence remains scarce [6]. Therefore, further studies remain to be carried out regarding the comprehensive analysis of ergonomics in the field of minimally invasive surgery (MIS) and mainly in robotic-assisted surgery, allowing us to precisely identify possible ergonomic deficiencies, design possible solutions and recommendations, and adapt training programs according to these needs.

Different techniques, both subjective and objective, have been used to assess ergonomics during robotic-assisted laparoscopic surgery to evaluate different physiological and cognitive factors. Some studies employ traditional methods of subjective assessment of the workload, both mental and physical, of surgeons during their robotic surgical activity, such as the SURG-TLX scale [7]. Dixon et al. compared the workload between robotic-assisted surgery and conventional laparoscopic surgery, concluding that robotic surgery with an open console system reduces ergonomic risk scores and cognitive strain in colorectal surgery relative to conventional laparoscopic surgery [8]. However, this subjective assessment method should be reinforced with objective ergonomic evaluation techniques.

On the other hand, photogrammetry studies make it possible to evaluate the surgeon’s posture during surgery to assess possible musculoskeletal risks. In the study by Brunner et al., the Cologne Ergonomic Measurement Setup for Robotic Surgery (CEMRobSurg) method was used to evaluate the surgeon’s posture during surgery with the Hugo^TM^ RAS System [9]. Different parameters related to ergonomic posture were evaluated in subjects with different levels of surgical experience, who were asked to perform standardized virtual robotic training exercises (Peg Board, Rope Walk, and Ring Walk) using the Hugo^TM^ RAS console. During the activity, the posture of the surgeon operating the console was recorded by means of a camera placed in a fixed position. Frames taken from the side of the console were evaluated using OpenPose, a machine learning model that estimates body pose in an image. However, this evaluation method is limited by the evaluation of the posture based on a 2D projection of the body segments, the detection of joint rotations, and possible occlusions. In the study, a risky positioning of the neck and elbow was observed in medical students and in the knee and hip region for expert surgeons.

Other studies used 3D capture systems such as the Xbox Connect camera to assess the surgeon’s posture during robotic practice [10]. Subsequently, they performed a correlation of the calculated results with ergonomic assessment methods such as Rapid Whole-Body Assessment (REBA) and Rapid Upper Limb Assessment (RULA). In this study, four robotic surgeries were observed with the da Vinci^TM^ Xi model for a minimum of 30 min each: two cholecystectomies, one partial colectomy, and one appendectomy. The results obtained for the RULA and REBA scores indicated a medium musculoskeletal risk with the recommendation that measures needed to be taken to improve surgeon ergonomics. However, as with most image-based postural analysis methods, this system is prone to occlusion problems in crowded environments such as operating rooms.

The evolution and miniaturization of sensors have allowed the increasing incorporation of wearable technology in ergonomic and physiological analysis in the surgical environment, which has facilitated objective solutions without interrupting the surgeons’ surgical practice and avoiding possible occlusion problems during surgery and without interfering with the sterile environment. Within these assessment systems, we highlight the systems for recording and analyzing movement based on inertial measurement units (IMU) [11], the systems for analyzing electromyographic (EMG) signals, and the level of stress through the examination of electrocardiogram (ECG) or electrodermal activity (EDA) signals.

Previous studies with these technologies concluded that the console could limit postures, causing static loads that have been associated with musculoskeletal symptoms for the surgeon’s neck, torso, and shoulders [11]. On the other hand, other studies indicated that laparoscopic practice presented more forearm muscle fatigue compared to robotic-assisted laparoscopic surgery [12]. Regarding the analysis of stress during RAS practice, it was observed that surgeons with better experience showed higher levels of stress than expert surgeons [9]. In previous studies focused on the comparison of ergonomics between laparoscopic surgery and RAS, our results indicated that robotic-assisted procedures showed better ergonomic outcomes for the lead surgeon compared to conventional laparoscopic surgery [13], using different wearable technologies to record the surgeons’ posture, muscle activity, EDA, and electrocardiographic signal during surgical practice.

The integration of artificial intelligence (AI) in healthcare has seen remarkable growth, expanding across various applications [14]. These AI algorithms utilize complex processes to uncover valuable insights hidden within data [15]. Among the diverse AI techniques, several algorithms facilitate the creation of predictive models. It is essential to distinguish these from machine learning models, which are associated with convolutional neural networks (CNNs) and deep learning models (DLMs). These latter models assess the outcomes of previous predictive models and learn from them [16].

In the realm of ergonomics during surgical practice, predictive models offer extensive possibilities for predicting risk situations that could affect a surgeon’s health, such as poor posture, muscle fatigue, or high stress levels. In previous studies, we successfully designed and implemented predictive models to identify high stress levels in minimally invasive surgery (MIS) by analyzing EDA data [17]. The linear models proposed in these studies were validated, demonstrating their potential to predict factors that can enhance surgeon health during operations. By predicting and mitigating these risky situations, we can potentially improve surgeons’ well-being and, consequently, the quality of surgical practice.

The main novelties of this study focus on improving the understanding of ergonomic risks during the practice of RAS, presenting a significant advance by correlating muscle activity with forced postures for the related joints. Similarly, we highlight the development of predictive models to enhance surgical training programs, improving the quality of the surgical procedure and patient care. Finally, these findings contribute to defining ergonomic guidelines for surgical practice, with the aim of reducing musculoskeletal risks and benefiting the surgeon’s health.

The article is organized as follows: Section 2 presents a brief description of the methodology applied in this study. Section 3 presents the main results obtained. Section 4 contains a discussion of the results extracted in the present study and the comparison with studies in the scientific literature. Section 5 summarizes the main conclusions of this research.

Consequently, the present study aims to advance the objective analysis of ergonomic challenges in RAS and to develop innovative solutions for their prevention in surgical practice. The relationship between the muscle activity of various muscle groups and the musculoskeletal risk of surgeons during RAS will be investigated. Likewise, this study aims to design and implement predictive models for the future prediction of musculoskeletal risk situations during MIS.

## 2. Materials and Methods

### 2.1. Setup

**Figure 1:**
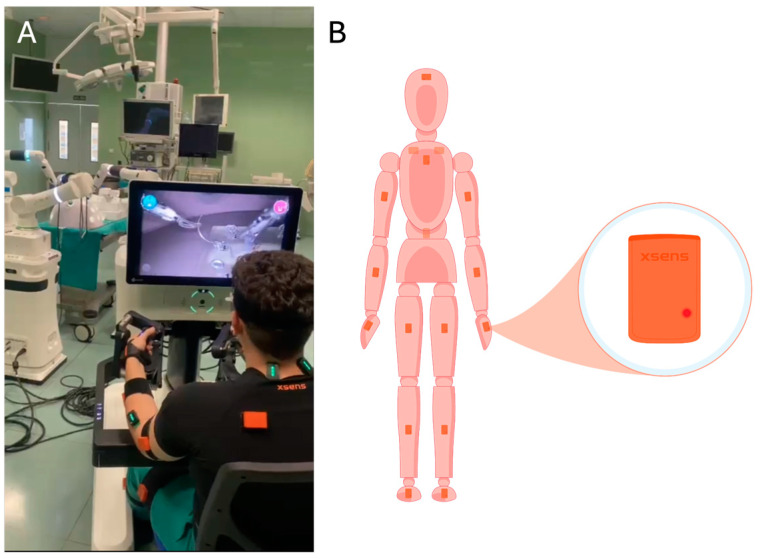
Surgeon using the Versius^TM^ robotic platform while wearing the EMG and motion analysis sensors (**A**). Body posture calibration of the Xsens motion analysis system and anatomical location of its inertial sensors for the analysis of the movement of each joint (**B**).

This study was carried out using the Versius^TM^ surgical platform (CMR Surgical; Cambridge, UK) for robotic-assisted surgical practice (Figure 1A). It is a modular robotic platform with an open console and three-dimensional vision.

All participants received a training session on the use of the robotic platform to learn the basics of its use, the handling of the controls, and its main functionalities in order to be able to perform safe surgeries.

To ensure the same ergonomic conditions for all surgeons, the height of the screen and console was adjusted before the study according to the height of their eyes and forearms.

This study involved three groups of surgeons: Surgeons experienced in laparoscopy (more than 100 laparoscopic procedures performed), surgeons experienced in microsurgery (more than 100 microsurgical procedures performed), and novice surgeons in both surgical disciplines (less than 10 surgical procedures performed).

### 2.2. Surgical Tasks

The participants carried out the following tasks with the robotic platform:

Peg transfer. The eye–hand coordination task consists of transferring rubber pieces in the form of elongated toroids from one pole to another by passing the piece from one hand to the other. Two fenestrated forceps were used for the dominant and non-dominant hands. A repetition was considered when the surgeon moved all three pieces to the three target poles. Participants were asked to complete two repetitions with a limit of 10 min.

Cutting. In this task, the surgeon was asked to cut two cutting templates consisting of a straight line with an arc in the center and a circular one. Half of each template was cut with the scissors of the dominant hand and the other half with the scissors of the non-dominant hand. A scissor and a Maryland dissector were used. A time limit of five minutes was set for each cutting template.

Labyrinth (needle passing). In this task, it is necessary to thread a needle through a circuit with holes in order and in different directions. This task aims to force uncomfortable postures, especially in the wrists, to evaluate the surgeon’s skill and the ease of returning to a correct posture. It is necessary to insert the needle with the dominant hand and remove it from the other side with the non-dominant hand. A needle holder was used in the dominant hand, and a Maryland dissector in the non-dominant hand. Participants were asked to complete the entire circuit within 10 min of the limit.

Suture. Finally, participants were asked to perform a suture on a simulated tissue model. To do so, they had to pass the needle through two specific entry and exit points and perform a double knot and two single knots in opposite directions. A needle holder was used in the dominant hand, and a Maryland dissector in the non-dominant hand. A time limit of ten minutes was set for the task.

### 2.3. Kynematic Recording Systems

The Xsens motion analysis system (Movella Inc.; Henderson, NV, USA) was used to record the surgeons’ body movements. This system consists of 17 inertial sensors to record the movements of the subject’s body segments in real-time, with a refresh rate of up to 60 Hz per sensor. The sensors were placed on the hands, forearms, arms, feet, legs, upper legs, lumbar and thoracic regions, shoulders, and head (Figure 1B).

In addition, the TRIGNO™ Avanti wireless EMG system from DELSYS (Natick, MA, USA) was used to record the surgeons’ muscle activity using electromyography (EMG) signals. This system has up to 16 sensors with a sampling rate of 2148 Hz. A trigger system was used to synchronize the recording between the Xsens and Delsys systems. The EMG signal was recorded bilaterally from the following muscle groups: upper trapezius, middle trapezius, deltoid, and brachioradialis, related to the activity of joints undergoing workload associated with laparoscopic procedures such as the neck, arms, and wrists, respectively [18]. EMG sensors were placed in each muscle group following SENIAM guidelines [19,20]. Before placing each sensor, the skin was cleaned by gently rubbing it with 70% isopropyl alcohol. The raw EMG signals were processed using a 20–450 Hz band-pass filter with a range of 11 mV (±5.5 mV). The filtered EMG signal was then smoothed with a 140 ms moving window, removing an offset from the signal, and calculated as a root mean square (RMS) value. To normalize the results for each subject, EMG values were presented as a percentage of maximal voluntary contraction (%MVC). MVC was performed separately for each muscle group just before each test by asking each subject to perform specific maximal contractions against a fixed resistance.

### 2.4. Data Analysis

Regarding the surgeon’s posture, the joints considered most representative in the analysis of the surgeon’s posture in robotic-assisted surgical practice were analyzed [18]: Shoulder, wrist, and neck flexion/extension; shoulder abduction/adduction and internal/external rotation; ulnar/radial deviation and pronation/supination of both wrists; axial flexion of the neck. Degree values were obtained for each joint and were compared with the EMG amplitude signal with regard to the three study groups.

#### 2.4.1. Body Posture Assessment

The rapid upper limb assessment (RULA) method [21] was used to assess the ergonomic risk of the surgeon’s body posture. RULA gives a musculoskeletal risk score for the posture of the neck, arms, and wrists, as well as an overall posture score for the subject. RULA scores were assessed only for the joints considered in the present study. Lower extremity analysis was not taken into account, as the surgeons were seated throughout the activity.

#### 2.4.2. Correlation Analysis

The sample set was assumed to be non-parametric due to the small number of subjects [22,23]. The Wilcoxon signed-rank test was used to evaluate significant differences for paired samples, like the relation between the anatomical structures (muscle activity and joint angles) with shared functionalities; meanwhile, the Mann–Whitney U Test was selected for those independent sample sets. Jamovi (Jamovi Project, 2024, Version 2.5) software platform was used for data and statistical analysis according to biostatistical literature [24,25,26].

#### 2.4.3. Predictive Analysis

From all the RULA data collected in this study, 15 datasets with 142,598 records were generated. The original datasets were then transformed by applying scale preprocessing and normalization techniques, resulting in 30 datasets. These pre-processed datasets were divided into 60 datasets: 80% of the data from each dataset for calibration and 20% of the data from each dataset for validation [27].

The scaled preprocessing technique allows each parameter to be described on a scale between 0 and 1 [28]. To do this, each value is subtracted from the minimum value and then divided by the interval between the maximum and minimum values (Equation (1)). In this way, all values of each RULA parameter are bounded between 0 and 1.

(1) $$Value_{new}\;=\;\frac{Value_{current}\;-\;min}{max\;-\;min}$$

where *value_new_* indicates the pre-processed value, *value_current_* represents the raw value from each dataset, *min* shows the minimum value for each parameter, and *max* indicates the maximum value for each parameter.

For the normalized preprocessing technique, the mean of each value is subtracted and divided by the standard deviation [28] (Equation (2)). This technique transforms the dataset into a more integrated and robust one with fewer redundancies.

(2) $$Value_{new}\;=\;\frac{Value_{current}\;-\;Value_{average}}{Value_{st.\;deviation}}$$

where *value_new_* indicates the pre-processed value, *value_current_* represents the raw value from each dataset, *value_average_* shows the average value for each parameter, and *value_st.deviation_* indicates the standard deviation value for each parameter.

#### 2.4.4. Artificial Intelligence

The free software WEKA (Waikato Environment for Knowledge Analysis, Hamilton, New Zealand, version 3.8.6) [29] was used to develop the predictive model. Two different AI predictive techniques have been applied to the calibration dataset to generate predictive models: Multiple Linear Regression (MLR) and Multilayer Perceptron (MLP).

MLR was applied as a linear predictive approach to the datasets. MLR shows the linear relationship between a dependent variable and several independent variables (Equation (3)). This technique arrives at a linear regression equation that can be used to predict future values. In this study, the M5 method of attribute selection was applied. This method cycles through the attributes, eliminating the one with the lowest standardized coefficient until no improvement in error estimation is observed. A peak value of 1.0 × 10^−4^ was applied [30].

(3) $$y\;=\;\sum_{i=1}^{n}\omega_{i}\;\cdot \;x_{i}$$

where *y* indicates the predicted value, *x_i_* represents the pre-processed value for each parameter, and *w_i_* shows the weights for each parameter.

MLP was applied as a predictive machine learning approach to datasets. MLPs are a type of artificial neural network model that are developed using neural organization principles [31]. Thus, different numbers of neurons are grouped into layers. The different layers can perform different transformations on their inputs. Signals travel from the first layer (the input layer) to the last (the output layer), possibly after passing through the layers several times. In the present study, the default configuration was used, with the learning rate equal to 0.3, the number of epochs equal to 500, the threshold equal to 20, and 30 nodes in the first hidden layer, 10 nodes in the second hidden layer, and 3 nodes in the third hidden layer.

To validate the generated models, a 10-fold cross-validation was carried out, in which the calibration dataset was divided into ten equally sized partitions. Each time a subset was tested, the remaining data were used to fit the model. The process was repeated sequentially until all subsets were tested. Therefore, all data were used for both calibration and validation. Although this method requires an analysis of ten replicates, it is a robust method [32]. Finally, the test dataset was used for external validation of the predictive models on the test dataset. The R^2^ coefficient was used to assess the goodness of fit of the prediction and for validation, according to the rules given by Colton [33], where R^2^ of 0 to 0.25 is considered as a poor to no relationship; 0.25 to 0.50 indicates a weak degree of relationship; 0.50 to 0.75 designates a moderate to good relationship; and 0.75 to 1 shows a very good to excellent relationship. The root mean square error (RMSE) was also used to validate the prediction results [34]. RMSE measures the difference between actual and predicted values. RMSE values of less than 0.05 are considered adequate [34].

## 3. Results

Seven surgeons participated in this study: three experts in laparoscopic surgery, two experts in microsurgery, and two novices in both specialties.

### 3.1. Kynematic Correlations

**Figure 2:**
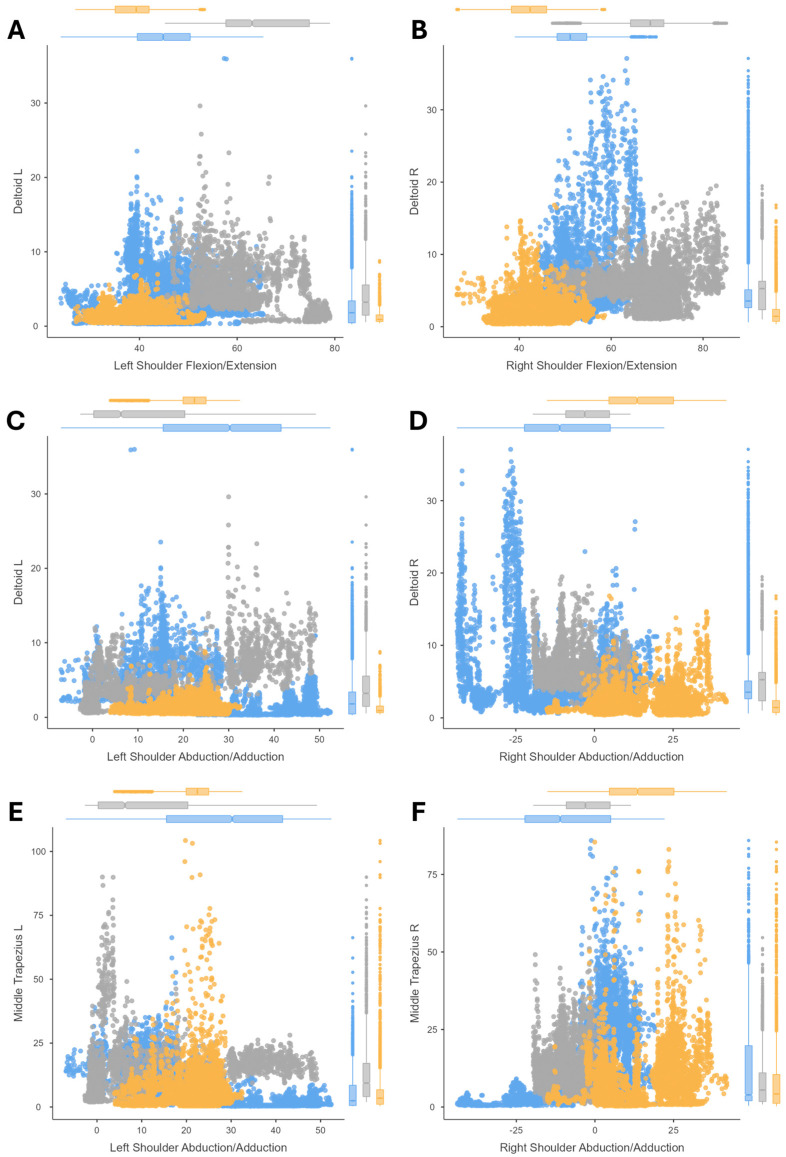
Correlation analysis for the cutting tasks. Results comparing the (1) deltoid amplitude signal and shoulder flexion/extension for the cutting task with the non-dominant hand (**A**) and with the dominant hand (**B**); (2) comparing the deltoid amplitude signal and the shoulder abduction/adduction for the cutting task with the non-dominant hand (**C**) and with the dominant hand (**D**); (3) and comparing the middle trapezius amplitude signal with the shoulder abduction/adduction for the cutting task with the non-dominant hand (**E**) and with the dominant hand (**F**). For three surgeons’ groups: novice surgeons (orange), expert surgeons in conventional laparoscopic surgery (blue), and expert surgeons in microsurgery (dark gray).

**Figure 3:**
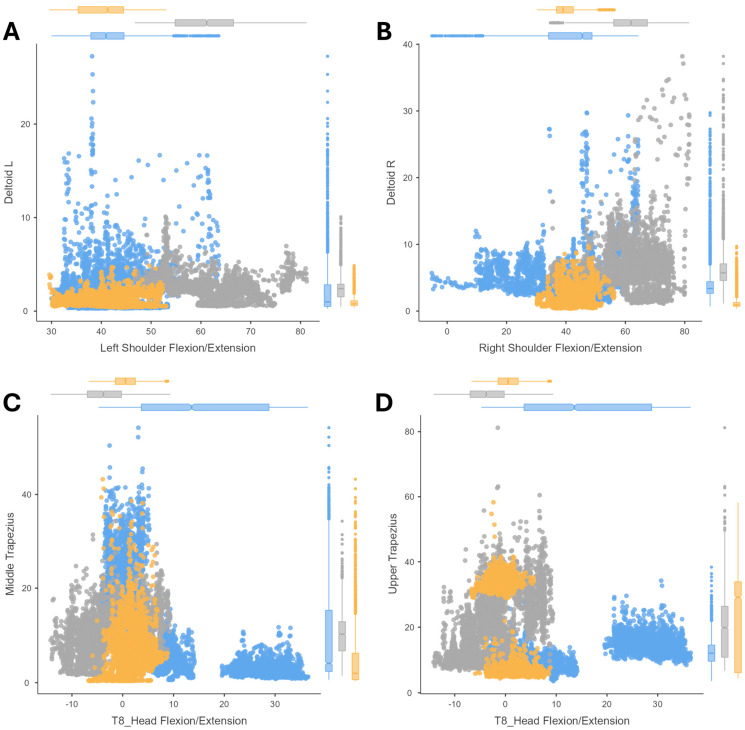
Correlation analysis for the peg transfer. Results comparing (1) the deltoid amplitude signal and the shoulder flexion/extension for the peg transfer task with the non-dominant hand (**A**) and with the dominant hand (**B**); (2) and results comparing the neck flexion/extension during peg transfer task with the middle trapezius (**C**) and upper trapezius (**D**) amplitude signal. For three surgeons’ groups: novice surgeons (orange), expert surgeons in conventional laparoscopic surgery (blue), and expert surgeons in microsurgery (dark grey).

**Figure 4:**
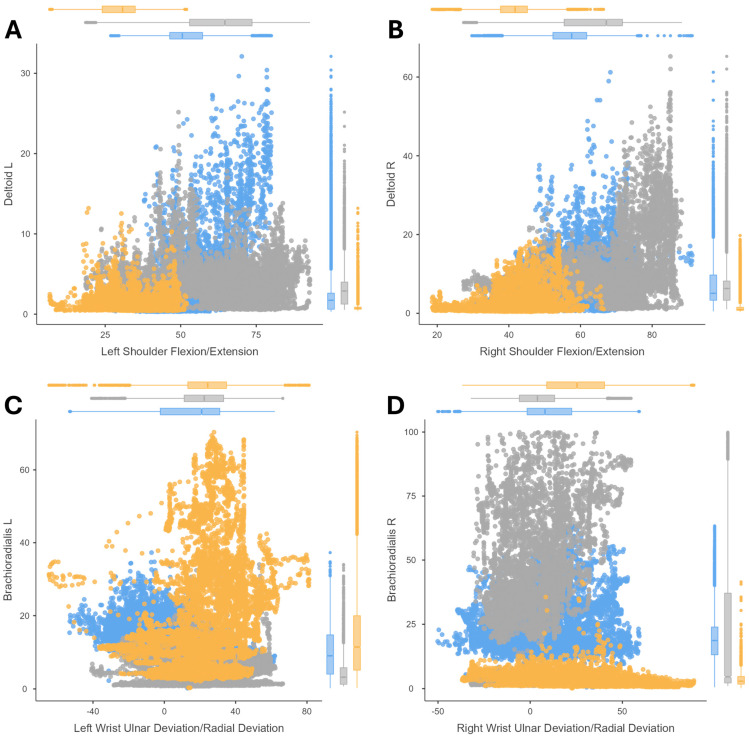
Correlation analysis for the labyrinth task. Results comparing (1) the deltoid amplitude signal and the shoulder flexion/extension. Results for the labyrinth task with the non-dominant hand (**A**) and with the dominant hand (**B**); (2) and comparing the brachioradialis amplitude signal and the wrist ulnar/radial deviation. Results for the labyrinth task with the non-dominant hand (**C**) and with the dominant hand (**D**). For three surgeons’ groups: novice surgeons (orange), expert surgeons in conventional laparoscopic surgery (blue), and expert surgeons in microsurgery (dark grey).

**Figure 5:**
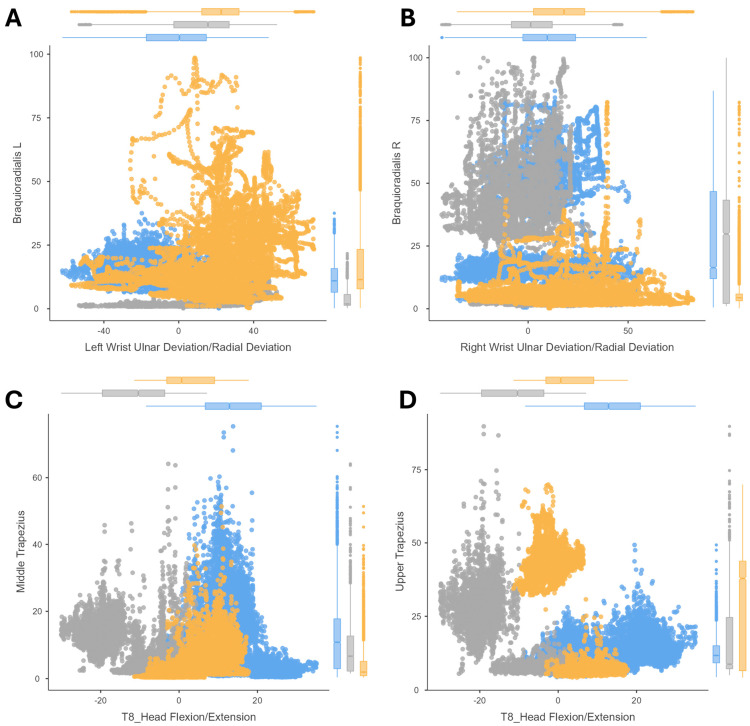
Correlation analysis for the suturing task. Results comparing (1) the brachioradialis amplitude signal and the wrist ulnar/radial deviation. Results for the suturing task with the non-dominant hand (**A**) and with the dominant hand (**B**); (2) and results comparing neck flexion/extension during the suturing task with the middle trapezius (**C**) and upper trapezius (**D**) amplitude signal. For three surgeons’ groups: novice surgeons (orange), expert surgeons in conventional laparoscopic surgery (blue), and expert surgeons in microsurgery (dark grey).

The correlations between the joint postures and their muscle activity (amplitude of the EMG signal) are shown by means of scatter plots. The results of cutting (Figure 2), peg transfer (Figure 3), labyrinth (Figure 4), and suturing (Figure 5) tasks are shown.

The most relevant results of the cutting task, due to the relationship between exercise needs and anatomical structures, are shown in Figure 2. Each pair of graphs consists of the same task performed with the non-dominant or dominant hand, the latter corresponding to the right hand for all subjects in the study. Attending to the shoulder flexion (Figure 2A,B), as a rule, all study groups experimented with an increase in the flexion with the right-handed task, with a corresponding increase in muscle activity. Shoulder abduction was evaluated considering both deltoid and middle trapezius activity. During the adduction, represented with negative values, an increase in the deltoid contraction (Figure 2D) against the hyperextension assumed by the middle trapezius (Figure 2F). On the other hand, the muscle activity during shoulder abduction was higher for the middle trapezius than the deltoid (Figure 2C,E), demonstrating the fundamental relationship between the activity of the middle trapezius and the abduction movement of the arms.

During the peg transfer task, each surgeon had to perform the same activity with both hands, represented by shoulder flexion in both arms (Figure 3A,B). However, to analyze neck flexion, both trapezius muscle groups were considered (Figure 3C,D). Comparing the shoulder flexion and extension shown during the cutting task (Figure 2A,B) in contrast to the peg transfer (Figure 3A,B), the overall muscle loading was slightly reduced for the latter task, showing a decrease in shoulder flexion of the dominant hand for the expert laparoscopists (Figure 3B). As for neck flexion (indicated by negative values), the upper trapezius experienced a higher level than the middle trapezius, being noticeable for both novice surgeons and expert microsurgeons.

In the case of the labyrinth task, the relationship between shoulder posture and deltoid muscle loading was analyzed, as well as the relationship between wrist posture and brachioradialis muscle activity (Figure 4). The results of the shoulder flexion analysis were similar between the peg transfer and the labyrinth tasks (Figure 3A,B and Figure 4A,B), with the posture being more strained in the microsurgery expert group of surgeons. Similarly, wrist activity recorded a large range of motion with increased muscle loading with the left hand in the novice group of surgeons (Figure 4C) and was even greater for the microsurgery expert group of surgeons with the dominant (right) hand compared to the other groups (Figure 4D).

The results for the suturing task show that, when passing the needle with the dominant hand and withdrawing it with the non-dominant hand, a similar behavior is presented for the labyrinth task (Figure 4C,D). The movement of the non-dominant wrist is irregular, and reports increased brachioradialis activity in the novice group of surgeons (Figure 5A), but the workload when passing the needle is higher in the experienced subjects (Figure 5B). Moreover, the activity of the middle and upper trapezius is slightly higher during the suturing task (Figure 5C,D) than during peg transfer (Figure 3C,D). However, the muscles respond in the same way, with activation of the middle trapezius during neck extension (Figure 5C) versus the upper trapezius during neck flexion (Figure 5D), showing how the activity of the medial trapezius can sometimes be increased by neck movements outside of neutral postures, except in hyperflexion.

Attending to the correlation analysis, assuming the null hypothesis, all *p*-values for the graphs above were less than 0.001, particularly for the Wilcoxon signed rank test where paired samples were analyzed, resulting in the absence of significant differences. However, the Mann–Whitney U Test for independent samples showed *p*-value = 0.004 in the middle trapezius during the peg transfer task comparing expert laparoscopists and expert microsurgeons’ results, as well as *p*-value = 0.007 in the upper trapezius for the same task between laparoscopic expert surgeons and novice surgeons, yet having sufficiently significant similarities. In all other cases, *p*-values were below 0.002.

### 3.2. Musculoskeletal Risk Assessment

Table 1 shows the results of the musculoskeletal risk assessment using the RULA method for the neck, arms, and wrists of the study groups during the performance of the training tasks. Ergonomics results are shown in mean values between 1 and 6, with a detrimental posture from 5 and upwards. In general, novice surgeons reflected better results with the arms than experienced subjects. Moreover, both novice surgeons and laparoscopic expert surgeons showed an ergonomic posture in the neck with values below 4. Finally, microsurgery expert surgeons were less careful about posture in the neck but moved further away than the rest of the experience groups from risky postures when working with their wrists.

### 3.3. Predictive Models

**Figure 6:**
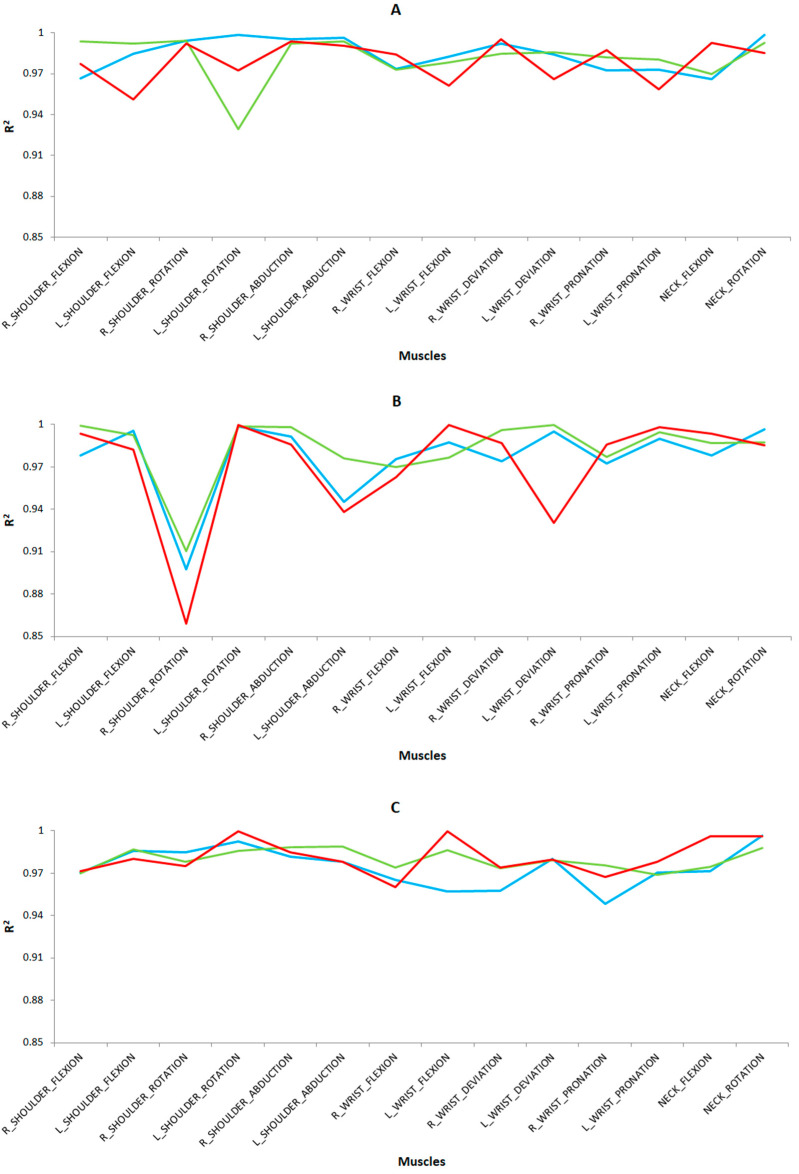

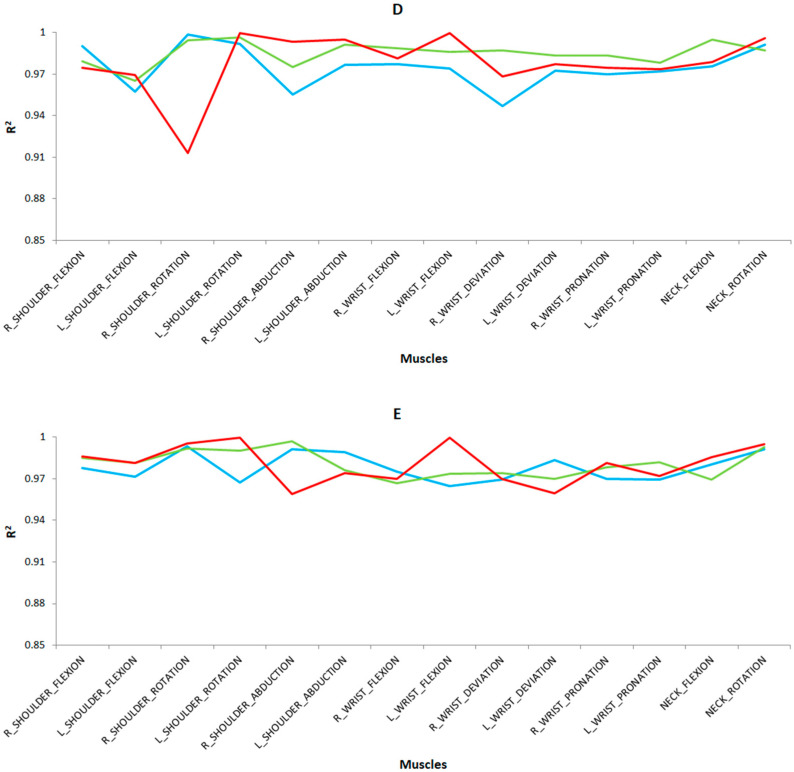
Results from the calibration dataset indicating the R^2^ values applying MLR as a predictive technique and scaling as a preprocessing technique in each case on five simulator tasks: (**A**) left cutting, (**B**) right cutting, (**C**) labyrinth, (**D**) peg transfer, and (**E**) suturing. For the three surgeon groups as a function of the surgical type and the surgeons’ level of expertise, being novice surgeons (red), expert surgeons on conventional laparoscopic surgery (blue), and expert surgeons on microsurgery (green).

The prediction results of the training dataset are shown in Figure 6 for the different tasks performed. In general, MLR showed slightly higher values than MLP for the R^2^ coefficient (R^2^ > 0.85) with a low RMSE error (RMSE < 0.05) for both cases. As for the preprocessing techniques, scaled achieved slightly higher values than scaled and normalized for the R^2^ coefficient. In general, the results obtained in the present study are satisfactory according to the standards given by Colton [33], reaching correlations (R^2^) close to 1 and RMSE close to 0 for all the movements studied.

It is worth highlighting the values of the R^2^ coefficient for the following body postures in each task and study group: for the left cutting task (Figure 6A), we highlight the right wrist deviation (R^2^ = 0.9955 and RMSE = 0.0035) for the group of novice surgeons; right shoulder rotation (R^2^ = 0.9941 and RMSE = 0.0059) for expert surgeons on microsurgery; and right (R^2^ = 0.9943 and RMSE = 0.0057) and left (R^2^ = 0.9984 and RMSE = 0.0006) shoulder rotation; right (R^2^ = 0.9951 and RMSE = 0.0039) and left (R^2^ = 0.9966 and RMSE = 0.0024) shoulder abduction; and neck rotation (R^2^ = 0.9987 and RMSE = 0.0003) for the expert group of laparoscopic surgeons. For the right cutting task (Figure 6B), we underline the left shoulder rotation (R^2^ = 0.9994 and RMSE = 0.0004), left wrist flexion (R^2^ = 0.9994 and RMSE = 0.0004), and left wrist pronation (R^2^ = 0.9983 and RMSE = 0.0007) for novice surgeons; right shoulder flexion (R^2^ = 0.9989 and RMSE = 0.0006), left shoulder rotation (R^2^ = 0.9987 and RMSE = 0.0008), right shoulder abduction (R^2^ = 0.9983 and RMSE = 0.0007), right (R^2^ = 0.9962 and RMSE = 0.0028) and left (R^2^ = 0.9997 and RMSE = 0.0007) wrist deviation, and left wrist pronation (R^2^ = 0.9947 and RMSE = 0.0053) for expert microsurgeons; and left shoulder flexion (R^2^ = 0.9957), left shoulder rotation (R^2^ = 0.9988 and RMSE = 0.0007), left wrist deviation (R^2^ = 0.9948 and RMSE = 0.0052), and neck rotation (R^2^ = 0.9964 and RMSE = 0.0026) for surgeons skilled in conventional laparoscopic surgery.

For the labyrinth task (Figure 6C), we highlight the left shoulder rotation (R^2^ = 0.9994 and RMSE = 0.0006), left wrist flexion (R^2^ = 0.9994 and RMSE = 0.0006), neck flexion (R^2^ = 0.9958 and RMSE = 0.0032), and rotation (R^2^ = 0.9961 and RMSE = 0.0029) for the novice group of surgeons.

For the peg transfer task (Figure 6D), we underline the left shoulder rotation (R^2^ = 0.9994 and RMSE = 0.0006), left shoulder abduction (R^2^ = 0.9951 and RMSE = 0.0039), left wrist flexion (R^2^ = 0.9994 and RMSE = 0.0006), and neck rotation (R^2^ = 0.9959 and RMSE = 0.0031) for the novice group of surgeons; right shoulder rotation (R^2^ = 0.9945 and RMSE = 0.0055) and left shoulder rotation (R^2^ = 0.9965 and RMSE = 0.0025) and neck flexion (R^2^ = 0.9961 and RMSE = 0.0029) for the expert group of microsurgeons; and right shoulder rotation (R^2^ = 0.9988 and RMSE = 0.0007) for the expert group of laparoscopic surgeons.

Finally, for the suturing task (Figure 6E), we emphasize the right (R^2^ = 0.9955 and RMSE = 0.0035) and left (R^2^ = 0.9994 and RMSE = 0.0006) shoulder flexion, left wrist flexion (R^2^ = 0.9994 and RMSE = 0.0006), and neck rotation (R^2^ = 0.9948 and RMSE = 0.0052) for the group of novice surgeons; and right shoulder abduction (R^2^ = 0.9972 and RMSE = 0.0028) in the expert group of surgeons in microsurgery.

**Figure 7:**
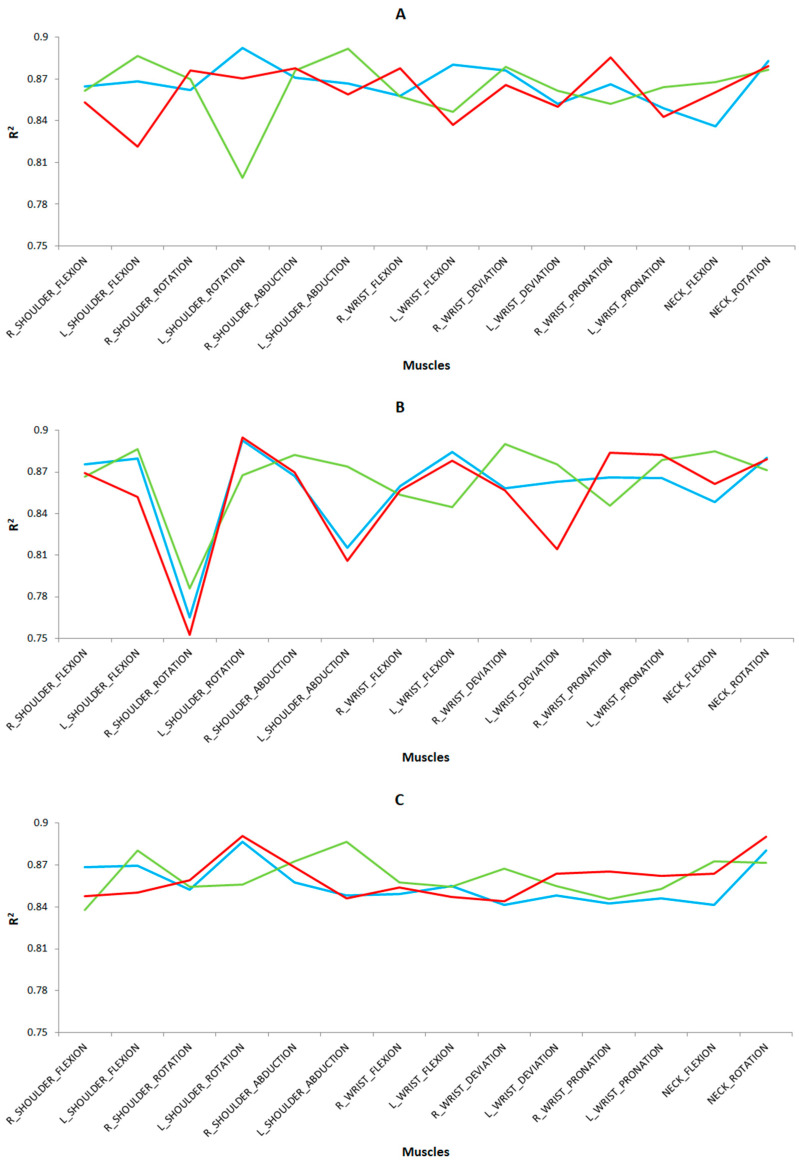

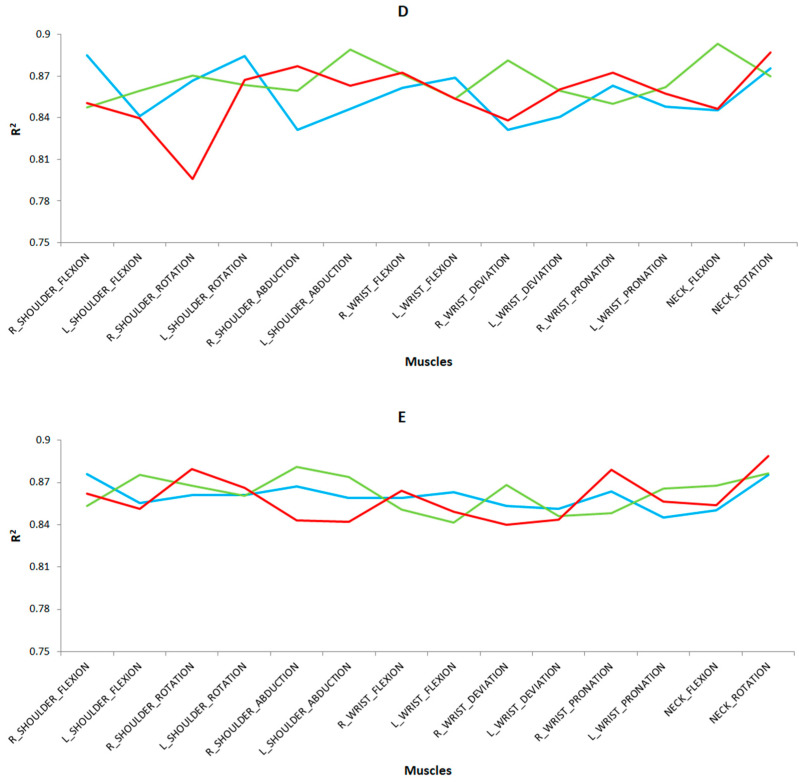
Prediction results from cross-validation on the calibration dataset indicating the R^2^ values applying MLR as a predictive technique and scaling as a preprocessing technique in each case on five simulator tasks: (**A**) left cutting, (**B**) right cutting, (**C**) labyrinth, (**D**) peg transfer, and (**E**) suturing. For the three surgeon groups as a function of the surgical type and the surgeons’ level of expertise, being novice surgeons (red), expert surgeons on conventional laparoscopic surgery (blue), and expert surgeons on microsurgery (green).

The prediction results of the cross-validation are shown in Figure 7 for the different tasks and study groups. In general, MLR showed slightly higher values than MLP for the R^2^ coefficient (R^2^ > 0.75) with a low RMSE error (RMSE < 0.05) for both cases. As for the preprocessing techniques, scaled achieved slightly higher values than scaled and normalized for the R^2^ coefficient. The results obtained in the present study are satisfactory according to the standards given by Colton [33].

Noteworthy are the R2 coefficient values for the following body postures in each task and study group: for the left cutting task (Figure 7A), we highlight the right wrist pronation (R^2^ = 0.8853 and RMSE = 0.0164), left shoulder flexion (R^2^ = 0.8864 and RMSE = 0.0142), and left shoulder abduction (R^2^ = 0.8919 and RMSE = 0.0051) for the novice surgeon group; and left shoulder rotation (R^2^ = 0.8924 and RMSE = 0.0046), left wrist flexion (R^2^ = 0.8805 and RMSE = 0.0260) and neck rotation (R^2^ = 0.8827 and RMSE = 0.0216), for the expert microsurgeons and expert laparoscopic surgeons. For the right cutting task (Figure 7B), we point out the left shoulder rotation (R^2^ = 0.8948 and RMSE = 0.0022), right (R^2^ = 0.8837 and RMSE = 0.0196), and left (R^2^ = 0.8823 and RMSE = 0.0224) wrist pronation for novice surgeons; left shoulder flexion (R^2^ = 0.8865 and RMSE = 0.0140), right shoulder abduction (R^2^ = 0.8823 and RMSE = 0.0224), right wrist deviation (R^2^ = 0.8902 and RMSE = 0.0068), and neck flexion (R^2^ = 0.8849 and RMSE = 0.0152) for expert microsurgeons; and left shoulder rotation (R^2^ = 0.8928 and RMSE = 0.0042), left wrist flexion (R^2^ = 0.8843 and RMSE = 0.0184), and neck rotation (R^2^ = 0.8804 and RMSE = 0.0262) for the expert group of laparoscopic surgeons.

For the labyrinth task (Figure 7C), we highlight left shoulder rotation (R^2^ = 0.8907 and RMSE = 0.0063) and neck rotation (R^2^ = 0.8901 and RMSE = 0.0069) for the novice surgeon group; left shoulder flexion (R^2^ = 0.8806 and RMSE = 0.0258) and abduction (R^2^ = 0.8867 and RMSE = 0.0136) for the expert microsurgeons; and left shoulder rotation (R^2^ = 0.8864 and RMSE = 0.0142) and neck rotation (R^2^ = 0.8805 and RMSE = 0.0260) for the expert laparoscopic surgeons.

For the peg transfer task (Figure 7D), we underline the neck rotation (R^2^ = 0.8869 and RMSE = 0.0132) for novice surgeons; left shoulder abduction (R^2^ = 0.8894 and RMSE = 0.0082), right wrist deviation (R^2^ = 0.8811 and RMSE = 0.0248), and neck flexion (R^2^ = 0.8931 and RMSE = 0.0039) for expert microsurgeons; and right shoulder flexion (R^2^ = 0.8852 and RMSE = 0.0166) and left shoulder rotation (R^2^ = 0.8847 and RMSE = 0.0176) for surgeons skilled in laparoscopic surgery. Finally, for the suturing task (Figure 7E), we highlight the neck rotation (R^2^ = 0.8888 and RMSE = 0.0094) for the novice surgeons and right shoulder abduction (R^2^ = 0.8812 and RMSE = 0.0246) for the expert group of microsurgeons.

**Figure 8:**
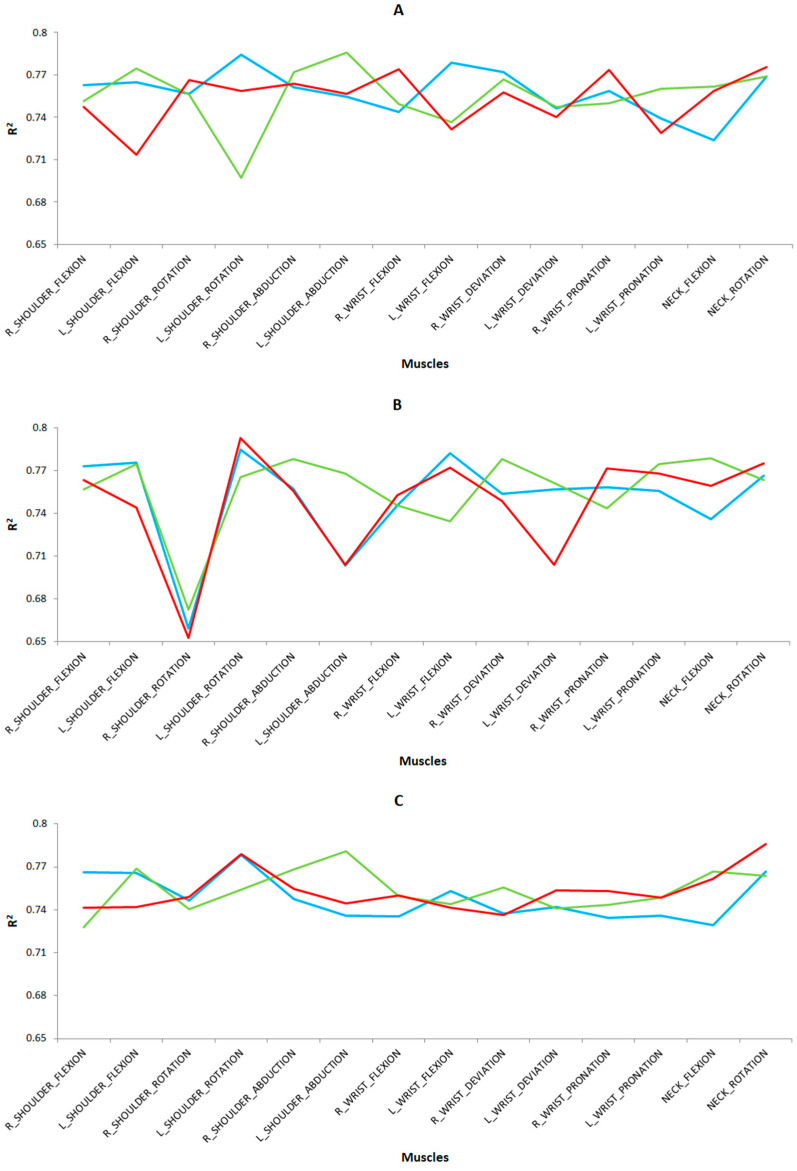

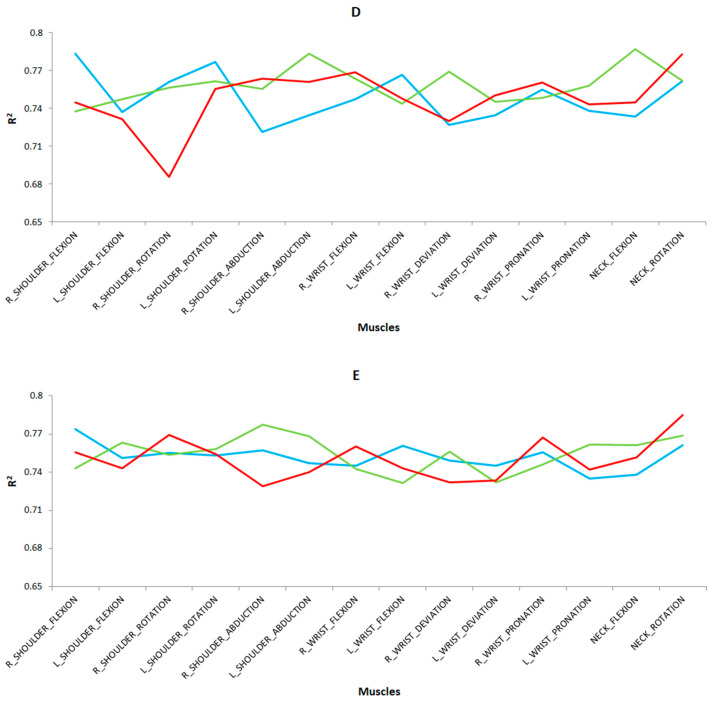
Results from the validation dataset indicating the R^2^ values applying MLR as a predictive technique and scaling as a preprocessing technique in each case on five simulator tasks: (**A**) left cutting, (**B**) right cutting, (**C**) labyrinth, (**D**) peg transfer, and (**E**) suturing. For the three surgeon groups as a function of the surgical type and the surgeons’ level of expertise, being novice surgeons (red), expert surgeons on conventional laparoscopic surgery (blue), and expert surgeons on microsurgery (green).

The results of the validation dataset are shown in Figure 8 for the different tasks and study groups. In general, MLR showed slightly higher values than MLP for the R^2^ coefficient with a low RMSE error (RMSE < 0.05) for both cases. As for the preprocessing techniques, scaled achieved slightly higher values than scaled and normalized for the R^2^ coefficient. Good to excellent correlations (R^2^ > 0.6) close to 1 and RMSE close to 0 were achieved for all the postures studied.

Of note are the R^2^ coefficient values for the following postures in each task and study group: for the left cutting task (Figure 8A), we highlight the right shoulder rotation (R^2^ = 0.7695 and RMSE = 0.0305), right wrist pronation (R^2^ = 0.7672 and RMSE = 0.0328), and neck rotation (R^2^ = 0.7848 and RMSE = 0.0224) for the novice surgeons; right shoulder abduction (R^2^ = 0.7772 and RMSE = 0.0228) and left shoulder abduction (R^2^ = 0.7681 and RMSE = 0.0319) and neck rotation (R^2^ = 0.7681 and RMSE = 0.0319) for novice surgeons; right (R^2^ = 0.7772 and RMSE = 0.0228) and left (R^2^ = 0.7681 and RMSE = 0.0319) shoulder abduction and neck rotation (R^2^ = 0.7686 and RMSE = 0.0314) for expert microsurgeons; and right shoulder flexion (R^2^ = 0.7738 and RMSE = 0.0262) for expert laparoscopic surgeons. For the right cutting task (Figure 8B), we point out the left shoulder flexion (R^2^ = 0.7757 and RMSE = 0.0243) and rotation (R^2^ = 0.7848 and RMSE = 0.0224), left wrist flexion (R^2^ = 0.7823 and RMSE = 0.0212), and neck rotation (R^2^ = 0.7664 and RMSE = 0.0336) for the novice group of surgeons; left shoulder flexion (R^2^ = 0.7745 and RMSE = 0.0255) and rotation (R^2^ = 0.7657 and RMSE = 0.0343), right (R^2^ = 0.7783 and RMSE = 0.0217) and left (R^2^ = 0.7681 and RMSE = 0.0319) shoulder abduction, right wrist deviation (R^2^ = 0.7782 and RMSE = 0.0218), left wrist pronation (R^2^ = 0.7747 and RMSE = 0.0253), and neck flexion (R^2^ = 0.7789 and RMSE = 0.0211) for the expert group of microsurgeons; and left shoulder flexion (R^2^ = 0.7757 and RMSE = 0.0243) and rotation (R^2^ = 0.7848 and RMSE = 0.0224), left wrist flexion (R^2^ = 0.7823 and RMSE = 0.0212), and neck rotation (R^2^ = 0.7664 and RMSE = 0.0336) for the expert group of laparoscopic surgeons.

For the labyrinth task (Figure 8C), we highlight the left shoulder rotation (R^2^ = 0.7787 and RMSE = 0.0213) and neck rotation (R^2^ = 0.7861 and RMSE = 0.0230) for novice surgeons; left shoulder flexion (R^2^ = 0.7686 and RMSE = 0.0314), right shoulder flexion (R^2^ = 0.7685 and RMSE = 0.0315), left shoulder abduction (R^2^ = 0.7807 and RMSE = 0.0203), and neck flexion (R^2^ = 0.7666 and RMSE = 0.0334) for expert microsurgeons; and right (R^2^ = 0.7663 and RMSE = 0.0337) and left (R^2^ = 0.7656 and RMSE = 0.0344) shoulder flexion, left shoulder rotation (R^2^ = 0.7784 and RMSE = 0.0216), and neck rotation (R^2^ = 0.7665 and RMSE = 0.0335) for expert laparoscopic surgeons.

For the peg transfer task (Figure 8D), right wrist flexion (R^2^ = 0.7684 and RMSE = 0.0316) and neck rotation (R^2^ = 0.7829 and RMSE = 0.0214) are notable for the novice group of surgeons; left shoulder abduction (R^2^ = 0.7834 and RMSE = 0.0217), right wrist deviation (R^2^ = 0.7691 and RMSE = 0.0309), and neck flexion (R^2^ = 0.7871 and RMSE = 0.0179) for the expert group of microsurgeons; and right shoulder flexion (R^2^ = 0.7832 and RMSE = 0.0216), left shoulder rotation (R^2^ = 0.7832 and RMSE = 0.0216), right wrist deviation (R^2^ = 0.7691 and RMSE = 0.0309), and neck flexion (R^2^ = 0.7871 and RMSE = 0.0189) for the group of experienced microsurgery surgeons, and left shoulder rotation (R^2^ = 0.7767 and RMSE = 0.0233) and left wrist flexion (R^2^ = 0.7668 and RMSE = 0.0332) for the expert laparoscopic surgeons.

Finally, for the suturing task (Figure 8E), we highlight the right shoulder rotation (R^2^ = 0.7695 and RMSE = 0.0305), right wrist pronation (R^2^ = 0.7672 and RMSE = 0.0328), and neck rotation (R^2^ = 0.7848 and RMSE = 0.0224) for novice surgeons; right (R^2^ = 0.7772 and RMSE = 0.0228) and left (R^2^ = 0.7681 and RMSE = 0.0319) shoulder abduction and neck rotation (R^2^ = 0.7686 and RMSE = 0.0314) for expert microsurgeons; and right shoulder flexion (R^2^ = 0.7738 and RMSE = 0.0262) for the expert group of laparoscopic surgeons.

## 4. Discussion

The advent of robotic-assisted surgery has brought a revolution in terms of the precision of surgical procedures, surgical maneuverability, and improved working conditions for the main surgeon, who can operate in a seated position with a three-dimensional view of the surgical field. However, there are still ergonomic constraints for surgeons that need to be addressed. Surveys reported that 56.1% of regularly practicing robotic surgeons continue to experience related physical symptoms or discomfort, mainly neck stiffness and finger and eye fatigue [5].

To carry out an exhaustive analysis of the surgeon’s ergonomic conditions, it is necessary to analyze several factors, such as posture [11], muscle activity [35], stress, or the subjective perception of the physical and mental load [7,36], among others. Previous studies have been able to analyze some of these factors, concluding that robotic-assisted surgery improves the surgeon’s ergonomic conditions compared to conventional laparoscopic surgery [13,37]. However, studies report that ergonomic deficiencies still exist in surgical robotics and that there is a need to improve the ergonomics of surgeon posture [5,10]. Although during robotic-assisted surgery the surgeon sits during the procedure, this posture leads to more back flexion compared to conventional laparoscopic surgery [3]. However, this flexion is usually less than 15 degrees and is therefore not highly detrimental from an ergonomic point of view.

Understanding how surgeons move during procedures allows us to improve and adapt robotic platform designs, better organize operating theatres, and consequently improve surgeon posture during surgical practice. Ergonomic guidelines, including training programs, for robotic surgery could be considerably improved if motion analysis is considered. To this end, methods for objective analysis of surgeon posture have evolved dramatically in recent years, from photogrammetry-based methods [38] to infrared camera systems [10] to studies completed using Xbox Connect Camera [39]. However, these camera-based systems are severely limited by occlusions and are therefore not suitable for complex and crowded environments such as an operating theater. Apart from the surgeon’s posture, it is essential to know and analyze the surgeon’s muscle activity to ensure proper ergonomics, as well as to ensure optimal use of instruments, which translates into precise movements during surgery. A previous study comparing RAS and conventional laparoscopic surgery found differences in muscle activation patterns [35], reporting that, in general, RAS requires lower levels of muscle activation in the neck and shoulder region. Wearable systems, such as the ones used in this work for recording the surgeon’s posture and muscle activity, offer highly versatile solutions for use in the operating theatre, unaffected by occlusions and respecting the surgeon’s freedom of movement and sterile conditions.

Regarding the cutting task, the increased muscle load shown in the right deltoid compared to the left deltoid in all study groups seems to be associated with increased joint range of shoulder flexion. This supports the potential evidence of a positive correlation between ergonomically incorrect posture and possible short-term muscle injury [40]. Shoulder abduction that occurs when seeking the proper posture during the cutting task was assessed with two different muscle groups (deltoid and middle trapezius) to analyze the level of involvement of each one. In this way, it was seen that shoulder abduction over-activates the middle trapezius, while it is shoulder adduction that relates to the deltoid, demonstrating that both muscle groups were worth analyzing with respect to this specific joint.

For the peg transfer task, shoulder flexion was significantly reduced compared to the cutting task. This may be due to not being required to seek complex postures in order to complete this basic task. The best adaptation to this task was by experienced laparoscopists, who significantly reduced the muscular load on the deltoid. The novice surgeons continued to maintain more appropriate postures than the expert microsurgeons, as the latter were probably not used to working so much with their arms but more with their forearms and wrists. For neck flexion, all subjects moved mainly in a range close to 0 degrees, with an increase in flexion in microsurgeons, possibly due to the habit of working with a microscope. Laparoscopists, on the other hand, occasionally experience neck extensions. In the case of the loading of the middle trapezius muscle in laparoscopists, it is in agreement with the results obtained in previous studies [41,42,43].

As for the labyrinth task, the degree of shoulder flexion was similar to that shown in the previous cutting and peg transfer tasks, although in this case, the muscular load progressively increased in the deltoids with respect to the cutting task. This task had an average of 30% MVC compared to the 10% MVC of the cutting task. This increased workload on the deltoids is accentuated in the case of the dominant hand due to the difficulty of completing the needle passage through the rings. The correlation between non-ergonomic posture and excessive muscle activation is notable in this task, as its duration was longer, and it presented an isometric load when maintaining an inadequate posture for a certain time. As for the brachioradialis muscle group, regardless of the position of the wrists, its muscular load was accentuated during the passing of the needle for the microsurgeons and when picking up the needle for the group of novice surgeons. Similarly, this increased muscle load may also be related to the stress and difficulty associated with the task for these groups with less experience in conducting these surgical maneuvers. These results are similar to those obtained in previous studies [13], in which novice surgeons showed increased muscle activity in several muscle groups, including the brachioradialis, during robotic-assisted surgery. It is important to highlight the importance of the brachioradialis muscle for the performance of surgical tasks, whose main action is to flex the forearm at the elbow joint and assist in pronation and supination of the forearm, which are crucial movements in laparoscopic and robotic surgical procedures.

As for the suturing task, with respect to the activation of the right brachioradialis, it is observed that while maintaining a range of wrist movement values similar to the maze task, as well as the same execution time limit, an increase in muscle load and the possible appearance of localized muscle fatigue is observed as a consequence. Therefore, it is shown that in this case, this increase is due to effort and not to body posture, perhaps because the task is considered the most complex and closer to real clinical practice.

In the case of neck flexion, it was observed that the progression of posture and the load suffered in this part of the body during all tasks concluded with the progressive reduction in neck extension by the laparoscopists as opposed to the tendency to increase the neck flexion in the microsurgeons group. Despite the time spent with incorrect postures during the analyzed tasks, muscle activity had hardly been observed to overload, with only a few values above 50% of the MVC in the upper trapezius muscle during neck flexion for the novice group of surgeons. Thus, we can conclude that robotic-assisted surgery allows a considerably optimal posture of the cervical spine. We believe this is due to the location of the screen and that it is only visible in 3D (through polarized glasses) if it is placed at eye level.

Taking into account the results obtained in previous studies [13,39] together with those presented in this work concerning the ergonomic risk analysis of surgeons during robotic practice (Table 1), it is concluded that there is a low ergonomic risk in neck and arm posture for novice surgeons and a medium ergonomic risk for experienced surgeons, although a medium-high ergonomic risk persists for the wrist posture in all study groups. These results, therefore, support the recommendation that surgeon posture needs to be improved during robotic-assisted surgery. The results obtained suggest that novice surgeons showed better ergonomic results compared to more experienced surgeons. Therefore, it appears that the influence of previous experience may be a determining factor in the ergonomic appropriateness of posture when performing robotic surgery. These results help us to identify possible ergonomic recommendations for each study group. In this case, microsurgeons should consider improving neck posture by avoiding cervical flexion, while novice microsurgeons should pay attention to the use of the non-dominant hand.

When it comes to adjusting the surgeon’s posture, one of the advantages of the RAS over conventional laparoscopic surgery is the adjustment possibilities offered by the platform with respect to the surgeon’s physical characteristics, allowing the height and proximity of the monitor, as well as the height of the controls and armrests, to be adopted. This allows surgeons to adapt their posture, mainly of the back, arms, and neck, to improve their ergonomics during surgery. It is important to comply with this adjustment in the use of the platform, especially for expert laparoscopists who often do not use the armrest correctly, which causes uncomfortable postures. The use of the clutch system is crucial to ensure correct postures during the entire surgical procedure, so intensive training in its use is essential.

Regardless of the different results between experience groups, there is hardly any progressive increase in muscle load when using the robotic platform, except for the arms. Therefore, it is recommended not to separate the arms from the armrests, avoiding abductions, although a learning period is necessary to acquire these skills and to completely avoid non-ergonomic arm postures, an objective that has already been achieved with the cervical vertebrae due to the design characteristics of the console.

Considering the results for the development and validation of the predictive model (R^2^ > 0.85 on the training dataset, R^2^ > 0.75 on the cross-validation, and R^2^ > 0.6 on the test dataset), the results are in agreement with those obtained in previous studies, showing a slightly higher R^2^ coefficient for MLR and the scaling preprocessing technique [17] for the training, cross-validation, and test datasets with values of R^2^ higher than 0.75. These results could be related to the variability of the surgeon’s surgical experience and the stress generated during the performance of surgical activities [43].

For the results of the training dataset, a high to excellent ratio (R^2^ > 0.75) was achieved in almost all cases, with expert laparoscopic surgeons being more accurate in the left cutting task, expert microsurgeons achieving the highest R^2^ values for most parameters in the right cut and peg transfer tasks, and novice surgeons being more reliable in the labyrinth and suture tasks. For the results of the cross-validation dataset, a high to excellent ratio (R^2^ > 0.75) was achieved in almost all cases, highlighting that expert microsurgeons were more reliable for the left and right cutting, peg transfer, and suturing tasks, and novice surgeons achieved the highest R^2^ values for most parameters in the labyrinth task. Furthermore, considering the results of the test dataset, as in the previous cases, a good to excellent ratio (R^2^ > 0.6) was achieved in almost all cases, with expert microsurgeons being more accurate in the left and right cutting and suturing tasks and novice surgeons achieving the highest R^2^ values for the labyrinth and peg transfer tasks.

As we have been able to observe in the results of this study, ergonomics in the field of surgical robotics continues to be an aspect that remains to be solved. Therefore, it would be advisable to include ergonomic recommendations in RAS training programs, promoting the reduction in musculoskeletal problems, the improvement of the surgeon’s health, and the consequent improvement of the quality of surgical practice. The predictive models that were developed shed light on the design of tools for the prevention of these musculoskeletal risk factors during the development of surgical activities. However, it would be necessary to include additional studies of more complex surgical procedures in order to obtain more accurate results.

Among the limitations of this study is the reduced number of participants. As this is a preliminary study, the number of participants in each study group was not remarkably high; there were only seven surgeons. For future work, efforts will be made to include a larger number of surgeons in each group, at least four in each group, as well as to include a group with experience in robotic surgery to obtain more conclusive and representative results. Similarly, this study only included basic laparoscopic surgery training tasks. It would be desirable to include more complex surgical tasks or procedures with a longer duration and different specialties, such as urology, gynecology, or general surgery, which are more representative of a real clinical situation. On the other hand, it would be of interest to increase the number of muscles studied since there are some, such as the triceps, that could have an influence on the development of surgical procedures. Similarly, it would be advisable to extend the range of AI algorithms analyzed in order to improve the prediction models, eliminating errors and biases. With accurate predictive models, it would be possible to reduce localized muscle fatigue, forced body postures, and other musculoskeletal risks, optimizing surgeons’ response and health. However, the advances presented in this study allow us to make steady progress in the search for a comprehensive analysis and better understanding of ergonomic conditions in minimally invasive surgery, as well as the development of innovative solutions to predict and improve surgeons’ health during surgical practice.

For future work, it is proposed to increase the number of participants, the muscle groups, the experience groups, and the type of robotic platforms used in order to obtain more conclusive results. Similarly, it would be desirable to include other assessment factors, such as physiological stress. In addition, the quality of surgical performance will be tested to see if musculoskeletal risk factors in surgical practice have an impact on surgical results.

## 5. Conclusions

This work contributes to the understanding of ergonomic risks in RAS, presenting a significant advance in the integration of wearable technology and the implementation of predictive models of musculoskeletal risks. The results of this work could significantly enhance surgical training programs in RAS, the design of ergonomic surgical tools, and improve surgeon health. These findings highlight the need for specific training programs based on the surgeon’s level of experience and comprehensive knowledge of ergonomic risks during surgical practice.

During this study, results have highlighted the overall positive correlation between prolonged maintenance of ergonomically inadequate posture during RAS and increased cumulative muscle activation that led to muscle fatigue and potential musculoskeletal problems. Specific relationships have also been identified, such as activation of the middle trapezius for both neck control and specific shoulder movements. In terms of experience groups, the ability of novice surgeons to work in RAS stands out compared to experienced surgeons. The values for laparoscopic expert surgeons are equally positive, in contrast to expert microsurgeons, who must adapt to a quite different surgical environment.

For the prediction models, the highest R^2^ coefficients were achieved by applying MLR as an artificial intelligence technique and scaling as a preprocessing technique, all the results reaching a good to excellent correlation ratio (R^2^ > 0.6). Considering the different groups and the different surgical tasks, the most accurate and reliable results were achieved by the expert group of microsurgeons for the cutting and suturing tasks and by the novice surgeons for the peg transfer and maze tasks. These results demonstrate the goodness and accuracy of the predictive models and are the starting point to reaching an exhaustive knowledge of the ergonomic risks of RAS.

## Figures and Tables

**Table 1:** Total RULA results related to every experience level, task, and joint recorded.

		Neck	Arms	Wrists
Experience	Task	Mean ± SD	Mean ± SD	Mean ± SD
Novel	Right Cut	3.17 ± 1.459	2.23 ± 0.447	4.92 ± 0.920
	Left Cut	3.37 ± 1.399	2.14 ± 0.361	5.66 ± 0.560
	Peg Transfer	3.32 ± 1.489	2.02 ± 0.147	4.67 ± 0.809
	Labyrinth	3.87 ± 1.418	2.43 ± 0.581	5.19 ± 0.831
	Suture	3.61 ± 1.375	2.41 ± 0.648	5.15 ± 0.834
Exp. Mic.	Right Cut	4.22 ± 1.319	3.00 ± 0.000	5.46 ± 0.604
	Left Cut	4.47 ± 1.143	3.21 ± 0.407	4.35 ± 0.880
	Peg Transfer	4.29 ± 1.277	2.99 ± 0.115	4.92 ± 0.736
	Labyrinth	4.67 ± 0.942	3.56 ± 0.561	4.79 ± 0.835
	Suture	4.79 ± 0.770	3.92 ± 0.288	4.59 ± 0.864
Exp. Lap.	Right Cut	3.58 ± 1.106	3.00 ± 0.642	5.21 ± 0.859
	Left Cut	3.11 ± 0.918	3.31 ± 0.798	5.20 ± 0.774
	Peg Transfer	3.44 ± 1.068	3.25 ± 0.824	4.83 ± 0.985
	Labyrinth	3.20 ± 1.154	3.41 ± 0.663	4.48 ± 0.890
	Suture	3.08 ± 0.905	3.30 ± 0.679	4.17 ± 0.801

Exp. Mic.: Expert surgeons on microsurgery. Exp. Lap.: Expert surgeons on laparoscopic.

## Data Availability

Data are available on request due to restrictions, e.g., privacy or ethics.

## References

[1] Hurley A.M., Kennedy P.J., O’Connor L., Dinan T.G., Cryan J.F., Boylan G., O’Reilly B. (2023). SOS save our surgeons: Stress levels reduced by robotic surgery. Gynecol. Surg..

[2] Rivero-Moreno Y., Echevarria S., Vidal-Valderrama C., Pianetti L., Cordova-Guilarte J., Navarro-Gonzalez J., Acevedo-Rodríguez J., Dorado-Avila G., Osorio-Romero L., Chavez-Campos C. (2023). Robotic Surgery: A Comprehensive Review of the Literature and Current Trends. Cureus.

[3] Gabrielson A.T., Clifton M.M., Pavlovich C.P., Biles M.J., Huang M., Agnew J., Pierorazio P.M., Matlaga B.R., Bajic P., Schwen Z.R. (2021). Surgical ergonomics for urologists: A practical guide. Nat. Rev. Urol..

[4] Kaplan J.R., Lee Z., Eun D.D., Reese A.C. (2016). Complications of minimally invasive surgery and their management. Curr. Urol. Rep..

[5] Lee G.I., Lee M.R., Green I., Allaf M., Marohn M.R. (2017). Surgeons’ physical discomfort and symptoms during robotic surgery: A comprehensive ergonomic survey study. Surg. Endosc..

[6] Müller D.T., Ahn J., Brunner S., Poggemeier J., Storms C., Reisewitz A., Schmidt T., Bruns C.J., Fuchs H.F. (2023). Ergonomics in robot-assisted surgery in comparison to open or conventional laparoendoscopic surgery: A narrative review. Int. J. Abdom. Wall Hernia Surg..

[7] Wilson M.R., Poolton J.M., Malhotra N., Ngo K., Bright E., Masters R.S. (2011). Development and validation of a surgical workload measure: The surgery task load index (SURG-TLX). World J. Surg..

[8] Dixon F., Vitish-Sharma P., Khanna A., Keeler B.D., on behalf of VOLCANO Trial Group (2024). Robotic assisted surgery reduces ergonomic risk during minimally invasive colorectal resection: The VOLCANO randomised controlled trial. Langenbecks. Arch. Surg..

[9] Brunner S., Müller D., Krauss D.T., Datta R.R., Eckhoff J.A., Storms C., Von Reis B., Chon S.H., Schmidt T., Bruns C.J. (2024). Cologne ergonomic measurement for robotic surgery (CEMRobSurg) using the Hugo™ RAS System. Surg. Endosc..

[10] Dwyer A., Huckleby J., Kabbani M., Delano A., De Sutter M., Crawford D. (2020). Ergonomic assessment of robotic general surgeons: A pilot study. J. Robot. Surg..

[11] Yu D., Dural C., Morrow M.M.B., Yang L., Collins J.W., Hallbeck S., Kjellman M., Forsman M. (2017). Intraoperative workload in robotic surgery assessed by wearable motion tracking sensors and questionnaires. Surg. Endosc..

[12] Armijo P.R., Huang C.K., High R., Leon M., Siu K.C., Oleynikov D. (2019). Ergonomics of minimally invasive surgery: An analysis of muscle effort and fatigue in the operating room between laparoscopic and robotic surgery. Surg. Endosc..

[13] Pérez-Salazar M.J., Caballero D., Sánchez-Margallo J.A., Sánchez-Margallo F.M. (2024). Comparative Study of Ergonomics in Conventional and Robotic-Assisted Laparoscopic Surgery. Sensors.

[14] Alowais S.A., Alghamdi S.S., Alsuhebany N., Alqahtani T., Alshaya A.I., Almohareb S.N., Aldairem A., Alrashed M., Bin Saleh K., Badreldin H.A. (2023). Revolutionizing healthcare: The role of artificial intelligence in clinical practice. BMC Med. Educ..

[15] Ávila-Tomás J.F., Mayer-Pujadas M.A., Quesada-Varela V.J. (2020). La inteligencia artificial y sus aplicaciones en medicina I: Introduccion y antecedentes a la IA y robótica. Aten. Primaria.

[16] Janiesch C., Zschech P., Heinrich K. (2021). Machine learning and deep learning. Elctron. Mark..

[17] Caballero D., Pérez-Salazar M.J., Sánchez-Margallo J.A., Sánchez-Margallo F.M. (2024). Applying artificial intelligence on EDA sensor data to predict stress on minimally invasive robotic-assisted surgery. Int. J. Comput. Assist. Radiol. Surg..

[18] Netter F.H. (2003). Atlas de Anatomía Humana.

[19] Hermens H.J., Freriks B., Disselhorst-Klug C., Rau G. (2000). Development of recommendations for SEMG sensors and sensor placement procedures. J. Electromyogr. Kinesiol..

[20] (2006). SENIAM Project (Surface ElectroMyoGraphy for the Non-Invasive Assessment of Muscles). http://www.seniam.org/.

[21] Kakaraparthi V.N., Vishwanathan K., Gadhavi B., Reddy R.S., Tedla J.S., Samuel P.S., Dixit S., Alshahrani M.S., Gannamaneni V.K. (2022). Application of the rapid upper limb assessment tool to assess the level of ergonomic risk among health care professionals: A systematic review. Work.

[22] Sheskin D.J. (2011). Handbook of Parametric and Nonparametric Statistical Procedures.

[23] Siegel S., Castellan N.J. (1988). Nonparametric Statistical for the Behavioral Sciences.

[24] Borysiuk Z., Blaszczyszyn M., Piechota K., Nowicki T. (2022). Movement Patterns of Polish National Paralympic Team Wheelchair Fencers with Regard to Muscle Activity and Co-Activation Time. J. Hum. Kinet..

[25] (2022). The Jamovi Project (Jamovi Version 2.3 Computer Software). https://www.jamovi.org.

[26] Varghese J.J., Aithal V.U., Sharan K., Devaraja K., Philip S.J., Guddattu V., Rajashekhar B. (2024). Comparison of Submental Surface Electromyography during Dry Swallow between Irradiated Head and Neck Cancer Survivors and Normal Individuals. Folia Phoniatr. Logop..

[27] Dietterich T. (1998). Approximate statistical tests for comparing supervised classification learning algorithms. Neural Comput..

[28] Oka M. (2021). Interpreting a standardized and normalized measure of neighborhood socioeconomic status for a better understanding of health differences. Arch. Public Health.

[29] Frank E., Hall M.A., Witten I.H. (2016). The WEKA workbench. Online Appendix for Data Mining: Practical Machine Learning Tools and Techniques.

[30] Caballero D., Caro A., Dahl A.B., Ersboll B.K., Amigo J.M., Pérez-Palacios T., Antequera T. (2018). Comparison of different image analysis algorithms on MRI to predict physico-chemical and sensory attributes of loin. Chemom. Intell. Lab. Syst..

[31] Wu X., Kumar V., Ross-Quinlan J., Ghosh J., Yang Q., Motoda H., Mclachlan G.J., Ng A., Liu B., Yu P.S. (2008). Top 10 algorithms in data mining. Knowl. Inf. Syst..

[32] Grossman R., Seni G., Elder J., Agarwal N., Liu H. (2010). Ensemble Methods in Data Mining: Improving Accuracy Through Combining Predictions.

[33] Colton T. (1974). Statistics in Medicine.

[34] Hyndman R., Koehler A.B. (2006). Another look at measures of forecast accuracy. Int. J. Forecast.

[35] Szeto G.P.Y., Poon J.T.C., Law W.L. (2013). A comparison of surgeon’s postural muscle activity during robotic-assisted and laparoscopic rectal surgery. J. Robot. Surg..

[36] Guzmán-García C., Sánchez-González P., Sánchez-Margallo J.A., Snoriguzzi N., Rabazo J.C., Sánchez-Margallo F.M., Gómez E.J., Oropesa I. (2022). Correlating Personal Resourcefulness and Psychomotor Skills: An Analysis of Stress, Visual Attention and Technical Metrics. Sensors.

[37] Dalsgaard T., Jensen M.D., Hartwell D., Mosgaard B.J., Jørgensen A., Jensen B.R. (2020). Robotic Surgery Is Less Physically Demanding Than Laparoscopic Surgery: Paired Cross Sectional Study. Ann. Surg..

[38] Sánchez-Margallo F.M., Sánchez-Margallo J.A. (2018). Assessment of Postural Ergonomics and Surgical Performance in Laparoendoscopic Single-Site Surgery Using a Handheld Robotic Device. Surg. Innov..

[39] Stefanidis D., Hope W.W., Scott D.J. (2011). Robotic suturing on the FLS model possesses construct validity, is less physically demanding, and is favoured by more surgeons compared with laparoscopy. Surg. Endosc..

[40] Schlussel A.T., Maykel J.A. (2019). Ergonomics and Musculoskeletal Health of the Surgeon. Clin. Colon Rectal Surg..

[41] Pérez-Duarte F.J., Lucas-Hernández M., Matos-Azevedo A., Sánchez-Margallo J.A., Díaz-Güemes I., Sánchez-Margallo F.M. (2014). Objective analysis of surgeons’ ergonomy during laparoendoscopic single-site surgery through the use of surface electromyography and a motion capture data glove. Surg. Endosc..

[42] Hubert N., Gilles M., Desbrosses K., Meyer J.P., Felblinger J., Hubert J. (2013). Ergonomic assessment of the surgeon’s physical workload during standard and robotic assisted laparoscopic procedures. Int. J. Med. Robot..

[43] Amairhanayagam A., Zecca M., Barber S., Singh B., Moss E.L. (2023). Impact of minimally invasive surgery on surgeon health (issue) study: Protocol of a single-arm observational study conducted in the live surgery setting. BMJ Open.

